# Experiences, influencing factors, and perceived outcomes from physical literacy interventions: a qualitative meta-synthesis

**DOI:** 10.1080/17482631.2026.2613973

**Published:** 2026-01-16

**Authors:** Jaime Barratt, Hannah Goss, Noah Erskine, Maeghan James, Clemens Töpfer, Klaus Pfeifer, John Cairney, Johannes Carl

**Affiliations:** a Department of Educational Studies, Brock University, St. Catharines, Ontario, Canada; b Health and Well-Being Centre for Research Innovation, The University of Queensland St Lucia, Queensland, Australia; c School of Health and Human Performance, Dublin City University, Whitehall, Dublin, Ireland; d School of Human Movement and Nutrition Sciences, and the Queensland Centre for Olympic and Paralympic Studies, The University of Queensland, St Lucia, Queensland, Australia; e School of Epidemiology and Public Health, University of Ottawa, Ottawa, Ontario, Canada; f Institut of Sports Science, Friedrich Schiller University Jena, Jena, Germany; g Department of Sport Science and Sport, Friedrich-Alexander University Erlangen-Nürnberg, Erlangen, Germany; h Deakin University, Institute for Physical Activity and Nutrition, School of Health and Social Development, Geelong, Australia

**Keywords:** Physical literacy, participant experiences, program outcomes, holistic development, physical literacy interventions

## Abstract

**Purpose:**

This study synthesised qualitative findings from physical literacy (PL) interventions, focusing on participants’ experiences and perceived outcomes. It also explored the key factors influencing these experiences and outcomes to inform future PL interventions.

**Methods:**

A secondary data analysis was conducted using a meta-aggregative approach to review qualitative studies from a previously published systematic review (PROSPERO; registration number: CRD42020188926). Studies were included if they: (i) reported results from a PL intervention, (ii) reported participant perceptions, and (iii) were published in English. Studies that reported only quantitative outcomes were excluded, and mixed-methods studies were eligible only if they contained extractable qualitative findings. The data were extracted, synthesised, and categorised using the Joanna Briggs Institute System for the Unified Management, Assessment, and Review of Information.

**Results:**

Following the ConQual approach, the overall quality, dependability, and credibility of the 12 included studies were scored as high. A total of 336 findings were aggregated into 95 sub-themes (i.e., categories), which were then categorised into 19 themes. As a result, three overarching themes (i.e., synthesised findings) were identified: program outcomes, factors influencing outcomes, and challenges with implementation.

**Conclusions:**

Our synthesis highlights the holistic, context-sensitive nature of successful PL interventions, filling a notable gap in the predominantly quantitative PL literature. Tailoring programs to participant needs, proactively addressing logistical barriers, and continuing to foreground participant perspectives remain crucial for enhancing effectiveness and refining future intervention strategies.

## Background

Worldwide adequate physical activity (PA) is an important protective factor against a myriad of common morbidities and all-cause mortality (Kaminsky et al., 2019; Lavie et al., 2019). Despite public health efforts, Williams and Gibson (2018) explain the low success rates and poor effectiveness of PA promotion initiatives to be a symptom of the framing and emphasis of PA being a determinant of health. An increasingly medicalised perspective towards PA tends to strip movement away from other potentially positive associations and unique meanings, including social connection, personal enjoyment, and cultural expression (Clarke & Adamson, 2023; Nicholls et al., 2018). In lieu of the aforementioned, PA initiatives should shift framing towards the determinants of PA opportunities (Williams & Gibson, 2018).

While PA research typically quantifies movement in terms of minutes, intensity, and frequency, physical literacy (PL) offers a more encompassing lens that situates human movement within a lifelong, multidimensional relationship with movement (Whitehead, 2001). PL has become an increasingly important framework for understanding and ultimately fostering sustained engagement in PA (Brown et al., 2020; Cairney et al., 2019; Clark et al., 2022). Defined as a dynamic, cyclical integration of physical, cognitive, affective, and social capabilities, PL positions movement as an innate, meaningful aspect of human life (Bailey, 2022; Edwards et al., 2018; Young et al., 2020). This perspective shifts the emphasis away from viewing PA behaviours solely as products of individual agency toward a more nuanced appreciation of the educational and social processes that shape them (Cairney et al., 2019). The cyclical nature of PL helps explain how specific competencies and contextual circumstances interact across the life course to influence both participation and health outcomes; for instance, how chronic health challenges may limit movement opportunities and motivation and curtail the acquisition of new skills (Cairney et al., 2019).

Evidence of PL intervention research now spans continents and delivery models. School-based programmes have embedded PL tasks into PE lessons, classroom learning, and active recess; community and after-school initiatives have been designed to help build confidence and competence; and family-focused workshops have supported parents to promote PL at home (Carl et al., 2022a). For example, in mainland China, a 10-week school-day programme that wove PL tasks into active recess periods improved children’s cardiorespiratory fitness, grip strength, and mathematics scores relative to usual practice (Zhang et al., 2023). A 25-country survey across Europe shows accelerating, though uneven, uptake of PL principles in intervention research (Carl, Barratt, et al., 2023). Further, qualitative work from Indonesia reveals that early-childhood teachers often equate PL with basic motor play, emphasising the need for targeted professional learning in these contexts (Friskawati, 2024). Together, these studies illustrate examples of the geographical reach and contextual diversity within existing PL intervention and research, reinforcing the value of synthesising evidence across settings.

Despite increasing scholarly interest, a universally endorsed definition of PL has yet to emerge (Young et al., 2020). Nevertheless, the major formulations converge on a core principle: sustaining meaningful engagement in movement across the lifespan. The International Physical Literacy Association (International Physical Literacy Association (IPLA), 2017) describes PL as the motivation, confidence, physical competence, knowledge, and understanding that enable individuals to value and take responsibility for lifelong PA. Australian Sports Commission's (ASC, 2025) Australian Physical Literacy Framework (APLF) goes a step further by explicitly integrating physical, psychological, social, and cognitive capabilities into a holistic learning process, while Sport England’s (2023) Physical Literacy Consensus defines PL as an individual’s “relationship with movement and physical activity throughout life” (para. 7). Whilst acknowledging the other definitions, we adopted the APLF definition, as it: (a) has been widely adopted across the world, (b) makes the social domain visible, that adds a relevant perspective for PL interventions, and (c) reflects the focus of the most recent discussions, also informing the Sport England consensus. Importantly, the commitment to lifelong, meaningful movement is a shared tenet across all definitions; thus, scholars are advised to adopt the wording that best suits their study context (Bailey, 2022).

In promoting a shared understanding and operationalization of PL, Young et al. (2023) cartography of controversies identified three particular ideologies that PL falls under: (1) as health-promoting PA, (2) as motor competence and (3) as phenomenological embodiment (p. 59). This current paper is situated within a pragmatist paradigm, where PL is positioned as a health-promoting cosmos (Young et al., 2023) that can improve health, and help individuals be more active across the lifespan. As such, our definition follows ASC’s (2025) definition, which defines PL as the integration of physical, psychological, social, and cognitive capabilities, behaviours, and skills that help us lead active lives. Among these capabilities exist a number of elements (e.g., connection to place, relationships) that contribute to lifelong engagement in PA (ASC, 2025). This definition in particular allows for a more broad synthesis in our study of participant experiences from PL interventions aimed at targeting at least one or more of the aforementioned capabilities.

### Participant voice in physical literacy intervention research

Throughout the last decade, a significant number of PL intervention studies have been published between the years of 2017 to 2022 (Carl et al., 2022a). This five-year window corresponds to the publication period of the systematic review from which the present secondary data analysis is derived and captures a concentrated surge in empirical PL intervention research following the release of foundational PL frameworks. Over this five-year period, previous reviews (Carl et al., 2022a, 2022b) have shown that the field of PL interventions cultivated quantitative, qualitative, and mixed-methods research designs. Despite the diversity in methodologies observed in these reviews, previous analyses have been biased toward quantitative methodologies (Edwards et al., 2018).

Against this background, a qualitative perspective on PL interventions is largely missing. Quantitative studies play an imperative part in fostering understanding surrounding PL, however, the post-positivist nature of relying to heavily on quantitative findings creates an inherent bias on valuing objectivity and replicability, often disregarding reflexivity, and importantly, participant voice (Leavy, 2017). While there are quantitative tools that facilitate the collection of participants’ perspectives through quantifiable measures, these tools seldom provide participants a genuine opportunity with the “ability to speak and be heard” (Leavy, 2017, p. 49). For example, the Canadian Assessment of Physical Literacy (CAPL-2; Caldwell et al., 2021), the Physical Literacy Assessment for Youth (PLAY) tools (Longmuir et al., 2018), and self-report scales like the Perceived Physical Literacy Instrument (PPLI; Sum et al., 2016) translate participant perspectives on competence, confidence, knowledge, and motivation, into numerical scores. Further, quantitative variables do not seem to sufficiently cover aspects of process evaluation, compared to qualitative studies (Leavy, 2017).

It is important to note that the need for representation of participant’s voices in the collective understanding of PL literature is not just a matter of theoretical interest. The presence of more qualitative methodologies that adequately consider participants’ voices, promotes active inclusion in the knowledge-building process (Watharow & Wayland, 2024), and provides researchers and policymakers with explicit first-hand accounts of participants’ experiences (Belton et al., 2022), hence allowing researchers to continue comprehensively improve the development, delivery, and evaluation of PL interventions.

### Aim and research questions

The purpose of this study was to synthesise qualitative findings from PL interventions, that were included in a previous systematic review (Carl et al., 2022b). This study aimed to answer the following research questions:

1. What were participants’ experiences and perceived outcomes after participating in a PL intervention?
2. What key factors of PL interventions influenced participants’ experiences and their perceived PL outcomes?

With consideration to the scarcity of qualitative investigations of participant experiences from PL interventions, these questions may help to inform researchers with valuable intimate and nuanced insights towards the construct of PL.

## Design and methods

The current study employed a secondary data analysis of a previously published study (Carl et al., 2022b), and was guided by the Joanna Briggs Institute (JBI) methodology for qualitative systematic reviews (Lockwood et al., 2020). A meta-aggregative approach was followed to allow for a review of qualitative evidence across different studies (Paterson, 2011). Meta-aggregation is an appropriate qualitative synthesis technique for the current secondary analysis, as it collected and aggregated data from previous studies, rather than re-interpreting the data to form misrepresentations and personal conclusions (Ruggiano & Perry, 2019). Importantly, this approach allows for the data to produce a generalisable finding that can help inform health policymakers and practitioners (Hannes & Lockwood, 2011a, 2011b).

The Joanna Briggs Institute System for the Unified Management, Assessment and Review of Information (JBI SUMARI; Munn et al., 2019), was utilised to conduct the meta-aggregative approach, as it allows for transparency and replication (Hannes & Pearson, 2011). The process in JBI SUMARI is as follows: i) screening for eligiblity, (ii) critical appraisal of studies, (iii) extraction of study findings, and (iv) meta-aggregation of findings (Hannes & Pearson, 2011).

### Search and selection of studies

The initial screening included interventions that (a) reported a PL intervention that was either theory-inspired (i.e., loosely profit from the assumptions of a theory, model, or framework) or theory-based interventions (i.e., explicit links between theoretical constructs and intervention content/techniques; Michie & Abraham, 2004), (b) were published in an academic journal or book section, and (c) were written in English language. For the present review, only qualitative studies from a larger systematic review of PL interventions (Carl et al., 2022a) were selected for inclusion in the current study. These qualitative studies were eligible for inclusion in the current if they met all of the following criteria: (i) reported primary results from an intervention explicitly framed around PL; (ii) presented first-hand participant perceptions (e.g., experiences, views on outcomes, reflections on implementation) captured via qualitative or mixed methods; and (iii) were published in English in a peer-reviewed journal. Studies were excluded if they (a) reported outcomes with no extractable qualitative data, (b) examined general physical-activity programmes without an explicit PL component, or (c) were conference abstracts, dissertations, or other grey literature.

This review has been registered in the International Prospective Register of Systematic Reviews (PROSPERO; registration number: CRD42020188926), and the search strategy and search terms are published in Carl et al. (2022a).

**Figure 1.**
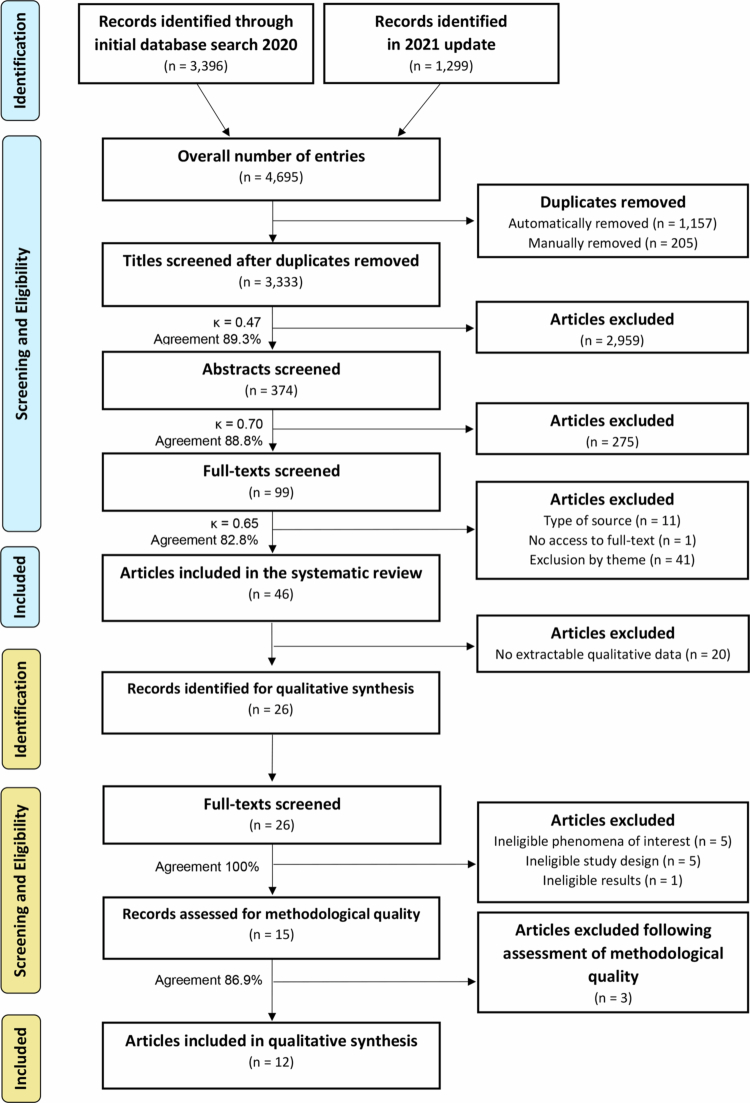
Articles included in the systematic review and secondary data analysis (Reporting Items for Systematic Reviews and Meta-Analyses (PRISMA) flow diagram; Page et al., 2021).

Of the 46 full-text records screened (Figure 1), 20 were excluded for not meeting one or more eligibility criteria, leaving 26 studies for the meta-aggregative synthesis. These 26 studies from Carl et al. (2022a) systematic review were qualitative studies identified and imported into the Joanna Briggs Institute System for the Unified Management, Assessment and Review of Information (JBI SUMARI; Munn et al., 2019) and were subsequently screened by two independent reviewers (JB & MJ) for assessment against the inclusion criteria. Qualitative studies met inclusion criteria if the study: (i) provided qualitative data eligible for qualitative synthesis, (ii) evaluated the interventions’ acceptability, feasibility, or effectiveness, and (iii) reported qualitative results that reflected either one or more elements of PL in line with the APLF (ASC, 2025).

### Assessment of methodological quality

Eligible studies were critically appraised by two independent reviewers (JB & MJ) for methodological quality using the standard JBI Critical Appraisal Checklist for Qualitative Research (Lockwood et al., 2015). This critical appraisal checklist asks ten questions relevant to the concept of dependability. It was predetermined that if a study did not meet either criteria for Q8 and/or Q9 (“Are participants, and their voices, adequately represented?” and “Is the research ethical according to current criteria or, for recent studies, and is there evidence of ethical approval by an appropriate body?”), it would be excluded without further review as these were previously identified as important to a high-quality methodological study (Keeping-Burke et al., 2020). Agreement rates were calculated and are presented in Table I. When discrepancies arose, the reviewers first reconciled them in a consensus meeting; any persisting disagreement was adjudicated by a third author (JC), whose decision was final.

**Table I.** Assessment of methodological quality of 15 studies.

Reference	Q1	Q2	Q3	Q4	Q5	Q6	Q7	Q8	Q9	Q10	Final decision
1. Bremer et al. (2020)	Y	Y	Y	Y	Y	N	N	Y	Y	Y	included
2. Campelo and Katz (2020)	Y	Y	Y	Y	Y	N	N	Y	Y	Y	included
3. Choi et al. (2021)	Y	Y	Y	Y	Y	Y	N	Y	Y	Y	included
4. Clutterbuck et al. (2020)	Y	Y	Y	Y	Y	N	N	Y	Y	Y	included
5. Demetriou et al. (2018)*	Y	Y	Y	N	Y	N	N	N	Y	Y	not included
6. Edwards et al. (2019)	Y	Y	Y	Y	Y	N	N	Y	Y	Y	included
7. Everley (2021)	U	Y	Y	Y	Y	N	Y	Y	Y	Y	included
8. Farias et al. (2020)	Y	Y	Y	Y	Y	N	N	Y	Y	Y	included
9. Lee et al. (2018)*	Y	Y	Y	Y	Y	N	N	N	Y	Y	not included
10. McLachlan et al. (2017)	Y	Y	Y	Y	Y	N	N	Y	Y	Y	included
11. Strobl et al. (2020)*	Y	Y	Y	N	N	N	Y	N	Y	N	not included
12. Telford et al. (2020a)	Y	Y	Y	Y	Y	N	Y	Y	Y	Y	included
13. Telford 2020b	Y	Y	Y	Y	Y	N	Y	Y	Y	Y	included
14. Usher (2018)	Y	Y	Y	Y	Y	Y	N	Y	Y	Y	included
15. Wright et al. (2020)	Y	Y	Y	Y	Y	N	N	Y	Y	Y	included
Agreement between reviewers (JB & MJ) %	94.73	100.0	100.0	89.47	94.73	90.52	26.31	84.21	94.73	94.73	

Note. Y yes, N no, U unclear. Studies with an asterisk did not fully meet the methodological inclusion criteria and were finally excluded from analysis (see also last column). Critical appraisal criteria for qualitative studies: Q1) Is there congruency between the stated philosophical perspective and the research methodology? Q2) Is there congruency between the research methodology and the research question or objectives? Q3) Is there congruency between the research methodology and the methods used to collect data? Q4) Is there congruency between the research methodology and the representation and analysis of data? Q5) Is there congruency between the research methodology and the interpretation of results? Q6) Is there a statement locating the researcher culturally or theoretically? Q7) Is the influence of the researcher on the research and vice-versa addressed? Q8) Are participants, and their voices, adequately represented? Q9) Is the research ethical according to current criteria or, for recent studies, and is there evidence of ethical approval by an appropriate body? Q10) Do the conclusions drawn in the research report appear to flow from the analysis or interpretation of the data?

### Data extraction

Using JBI SUMARI (Munn et al., 2019), qualitative data were extracted from each article by one independent reviewer (JB). All qualitative data were extracted from the full-text primary studies already included in Carl et al. (2022a systematic review; no additional search for new studies was conducted. The data extracted included details about the: phenomena of interest (i.e., study aims), participant information (e.g., demographics), study setting, study methods, and a description of the study’s primary findings. Information within the primary findings column were paraphrased from the original study’s results.

### Data synthesis and meta-aggregation

Qualitative research findings were amalgamated using JBI SUMARI (Munn et al., 2019) using the meta-aggregation approach (Lockwood et al., 2015). The first step in meta-aggregation involved the extraction of relevant findings, which were the main interpretations by researchers that were often presented as themes. To account for author variations and syntheses of results (e.g., studies reporting on acceptability and feasibility), similar to Sandelowski and Leeman (2012), a line-by-line review was conducted, focusing on results and discussion sections in each study. Using JBI SUMARI (Munn et al., 2019), findings were highlighted and extracted from each study, and then exported to a Microsoft Excel sheet to further organise the data.

The second step involved grouping the findings (i.e., author commentary and participant quotes) into categories– or what we refer to in this study as “sub-themes”. These were initially identified by original authors (JC & JB), and thereafter reviewed by a third author (HG) to avoid researcher bias and ensure interpersonal validity in the sub-themes identified (Lachal et al., 2017). Grouping continued until meaning saturation (i.e., analytic completeness whereby no new categories or interpretive insights emerged) was reached (Rahimi & Khatooni, 2024).

Typically, in meta-aggregation the next step would be to synthesise these categories into final synthesised statements (Lockwood et al., 2015), however, due to the hierarchical nature of PL (Cairney et al., 2019) and the interest in identifying outcomes in each PL domain, the next step involved categorising sub-themes into broader themes.

Lastly, these broader themes were given a label semantically representing the overall meaning of categories– which in this study we refer to as “overarching findings.” These overarching findings aligned with the aim of the study; to identify the outcomes after participating in a PL intervention, and key factors of that influenced participants’ experiences and their perceived PL outcomes.

### Assessing confidence in the findings

The final synthesised findings were graded within JBI SUMARI (Munn et al., 2019) according to the ConQual (i.e., confidence qualitative evidence) approach for establishing confidence in the output of qualitative research synthesis (Munn & Porritt, 2014). Following the ConQual approach, credibility was established by JB, assigning each “finding” a level of credibility: unequivocal (finding accompanied by an illustration), equivocal (finding lacking clear association with an illustration), or unsupported (finding not supported by data; Munn & Porritt, 2014). Once each finding was assigned a level of credibility, the researcher (JB) assigned an overall score to the finding of either high, moderate, low, or very low, depending on how many findings are assigned each level (Munn & Porritt, 2014).

## Results

### Study inclusion

#### Methodological quality

Of the 26 qualitative intervention studies identified from the parent review, 11 were excluded at this stage due to ineligible phenomena of interest (*n* = 5), ineligible study design (*n* = 5), or ineligible results (*n* = 1), leaving 15 studies for methodological appraisal. Upon assessing the methodological quality of the selected 15 studies, only 12 met the inclusion criteria (i.e., study did not meet either criteria for Q8 and/or Q9; See Figure 1). All these studies included were qualitative in design and included findings relevant to the systematic review objective and questions. Table I summarises the methodological quality of the 15 studies selected for the current article. Most of the criteria were addressed in the included studies (*n* = 12). The two criteria addressed least frequently were Q6 (“Is there a statement locating the researcher culturally or theoretically?”) and Q7 (“Is the influence of the researcher on the research, and vice versa, addressed?”), where only two studies (Choi et al., 2021; Usher, 2018) met the criteria for Q6, and five studies (Everley, 2021; Strobl et al., 2020; Telford et al., 2020a, 2020b) Q7.

#### Characteristics of included studies

The 12 studies included in this study were published between 2017 and 2021, and examined a range of target samples, including children and youth aged 5 to 12 (Bremer et al., 2020; Clutterbuck et al., 2020; Everley, 2021; Farias et al., 2020; Telford et al., 2020a; Usher, 2018), university lecturers (Choi et al., 2021), older adults (Campelo & Katz, 2020), physical education/primary school teachers (Telford et al., 2020b; Wright et al., 2020), parents and physiotherapists (Clutterbuck et al., 2020), and early childhood educators (McLachlan et al., 2017). Detailed demographic descriptors for every study (e.g., age ranges, sex, sample size, etc) have been published in an earlier systematic review (Table II in Carl et al., 2022a).

**Table II.** Summary of studies included in the meta-synthesis.

Study	Phenomena of interest	Setting	Participant characteristics	Methods	Description of main results
Bremer et al. (2020)	Examined feasibility and acceptability of a PL programme.	Afterschool programme in Hamilton, Ontario, Canada.	*N* = 7 Programme Leaders (6 females, 1 male; mean age 31.1; Ethnicity 3 White, 2 Black, 2 Other; average of 2 years experience as Programme Leader, and 4 reported previous PA training, while 3 reported none).	Experimental design. Programme leaders provided programme feedback via 5 open-ended questions post-intervention. No analyses were required as responses were presented verbatim.	Programme leaders perceived the programme to be effective in engaging children in FMS and PA. Programme activities were mostly acceptable by participants, however, leaders requested appropriate resources to fulfil programme expectations (e.g., space, equipment).
Campelo and Katz (2020)	Examined older adults’ experiences and changes in their PL, during an exergame programme.	Community and independent-living centres in Calgary, Alberta, Canada.	*N* = 15 participants (ET = 6, CT = 4, NT = 5) attended post-intervention focus groups. 10 identified as female. The average age was 73.53 years. All participants were independent community-dwelling older adults; two (CT, *n* = 1; NT, *n* = 1) were residents in an independent-living facility.	Focus groups (*n* = 3) from a larger study were analysed using an interpretive descriptive approach. Data were analysed using both an inductive and deductive approach. Data were mapped into four domains of PL (physical, psychological, cognitive, social), and raw data was categorised into over-arching themes.	Participants perceived the programme and use of wearable technology as intrinsically and extrincially motivating to engage in PA, despite the technological challenges some experienced. The social context of the programme was percieved to improve some elements of psychological and social domains of participants’ PL.
Choi et al. (2021)	Examined the influence of a SE–CPD programme on University lecturers’ conceptualisation and operationalization of PL.	University in Hong Kong.	University lecturers (PE specialists); homogenous sample of 8 (6 male, 2 female) from variety of academic, PA, and sport coaching backgrounds.	Post intervention semi-structured interviews followed by focus group interview. All data was transcribed verbatim in Cantonese, then analysed in English using grounded theory, and open, axial, and selective coding.	The pedagogy of the programme contradicted the classroom normative assessment, and negatively impacted teacher’s receptivity to the Sport Education pedagogical model. However, teachers perceived the programme beneficial to students’ PL.
Clutterbuck et al. (2020)	Examined participants’ perceived acceptability and effectiveness of the intervention: Sports Stars, on their PL.	Cerebral Palsy-Choice, Passion, Life in 7 locations across one state in Australia.	*N* = 8 physiotherapists responded to the perspectives survey post-intervention. Most (*n* = 5) were female and almost all had >4 years experience working with children with disabilities.	Exploratory study, collected data via open-ended surveys. Post-intervention, physiotherapistis and parents responded to open-ended questions regarding intervention participation, PL, and acceptability.	Sports Stars was generally perceived as an acceptable, enjoyable and effective programme for school-aged children with CP’s PL. Overall, the programme was perceived to improve children’s PL; specifically sport-specific activity, cognitive, social, and psychological competencies.
Edwards et al. (2019)	Explored the perceived impact that a professional development programme had on primary school teachers’ knowledge and operationalization of PL.	Medium primary schools from different SES across South Wales, United Kingdom.	*N* = 3 teachers (1 from City School [23 years experience], 2 from Urban School [3 and 20 years experience, respectively). 1 Urban School teacher was a PE specialist.	Semi-structured interviews pre- and post-intervention. Transcripts were analysed using both an inductive and deductive thematic approach. Data was categorised into two overarching themes: knowledge and operationalization of PL.	The intervention was perceived as effective at increasing teachers’ understanding of the PL definition, PE-specific knowledge, use of ipsative assessment, and confidence in operationalizing PL. Factors from the interventions which influenced positive outcomes included the use of workshops, use of differentiated learning tasks, and the needs assessment phase.
Everley, (2021)	Evaluated the impact of The English Football Association’s active literacy through storytelling programme on primary school girls’ social learning (in relation to PL).	Meadowfield school and forest school in Mid Sussex area of England, United Kingdom.	Meadowfield school: *N* = 8 (age 5/6 years, Year 1). Forest school: Year R: *n* = 4 (aged 5 years) Year 1: *n* = 4 (aged 5/6 years) Year 2: *n* = 3 (aged 6/7 years) Girl leaders: *n* = 6 (Year 6, aged 10/11 years).	Case study approach where data was collected using 3 methods: (I) Participant drawing of engagement before and after programme, (II) Individual interviews to discuss drawings, (III) Group interview to share experiences. Interviews were analysed thematically and sensitised by concepts of PL.	The interactive, social dynamic with differing ages in the programme influenced participants’ development of social capital, leadership, and relationships between students. The programme promoted participants’ communication, planning and organisation, and overall leadership skills.
Farias et al. (2020)	Explored students’ attitudes and perceptions of subsequent and current PL.	Sport Education programme in a K-12 School in Northern Portugal.	*N* = 24 7^th^ grade students (nine girls, 15 boys, ages 15–17 years old). One girl and one boy declined participation. One teacher, (45 years, 18 years experience), who was the class director and art teacher for grade 7−9 students.	Follow-up of a 2013-14 action research project. Multi-step qualitative data collection was informed by autobiographical memory theory: (1) project setting tour, (2) retrospective survey, (3) content analysis of project PE lessons, (4) semi-structured focus groups, and (5) individual, one-on-one semi-structured interviews. Data were analysed using the constant compartive method.	The programme improved participants’ PL (sense of self, movement competence, confidence, and engagement in social interactions), which in turn, developed a positive attitude toward PE and a revived sense of positive sport culture. Further, following participation in the project, students reported a commitment to pursue meaningful PA experiences. The factors associated with changes included the use of team dynamics (cooperation, team membership, competition), and the use of role-playing.
McLachlan et al. (2017)	Explored impact of the Jumping Beans programme on children’s PA, PL, and resilience.	Childcare centres in low SES areas (*N* = 4) in New Zealand.	*N* = 18 teachers (16 females; 2 males). *n* = 6 held Diploma of Teaching ECE; *n* = 5 Bachelor of Education; *n* = 4 a Bachelor of Teaching ECE; *n* = 2 a Graduate Diploma of Teaching ECE, *n* = 1 a Graduate Diploma of Teaching (Primary). The average amount of experience was 7.8 years.	Study reported data from of a larger, mixed-method intervention. Qualitative data was collected via semi-structured interviews with teachers. Researchers conducted content and thematic analyses using the constant comparative method.	The programme expanded teachers’ range of teaching practices, improved their awareness surrounding the importance of PA, gained knowledge, skills, and confidence to implement more PA and develop children’s FMS. One of the key factors in these changes was the distribution of resources and the professional learning workshop.
Telford et al. (2020a)	Examined the impact of the PEPL approach on primary school students’ PL (MVPA, FMS, attitudes toward PA, and self-perceptions).	Public Primary Schools (*N* = 14) in the suburbs of Melbourne, Australia.	*N* = 318 grade 5 students (51% girls). IG pre: *n* = 147 (mean age: 10.37) IG post: *n* = 126 (mean age: 11.13) CG pre: *n* = 156 (mean age: 10.41) CG post: *n* = 137 (mean age: 11.14)	This study was a pragmatic stratified cluster randomised controlled trial. Focus group interviews were conducted with primary school teachers, and data was analysed using a 5 step procedure outlined by Côté et al., (1993).	The programme developed students’ confidence, awareness of self-competencies, motivation to engage in PE, and improved level of enjoyment. Factors associated with changes included the characteristics of the PEPL coach, types of PE lessons and activities that were implemented, and the social context and ability to engage in teamwork.
Telford et al. (2020b)	Examined the percieved acceptability and impacts of the PEPL approach on teacher and school outcomes.	Public Primary Schools (*N* = 14) in the suburbs of Melbourne, Australia.	Classroom teachers: *n* = 8 Principal/Assistant Principals: *n* = 7 Specialist PE teachers: *n* = 3 PEPL coach: *n* = 1	Data was collected through interviews with Teachers, and Principals. Transcripts were thematically analysed using a 5 step procedure outlined by Côté et al., (1993).	The programme was percieved to improve teachers’ practice (i.e., implement PE in weekly activities), improve confidence to deliver PE, increased recognition of the importance of activity breaks, and improved student classroom behaviour. Factors associated with changes included the use of a PEPL coach, PE professional development, intervention flexibility, and the structured design of the programme.
Usher (2018)	Anlayzed adult participants’ perspectives of the MLP to reveal the effectiveness on young childrens’ affective, PL, SEWB, and CS development.	ECC and National Rugby League clubs in Gold Coast, Queensland, Australia.	*N* = 38 adults (included parents, centre directors, centre staff, programme staff, coaching staff; 90% were female). *n* = 3 were parents of participating children. More than 50% attended MLP at NRL; 32% at ECC, and 13% elsewhere.	Interpretive qualitative design. Data was collected first via one-on-one open-ended interviews with participants, and subsequently an open-ended online survey. An inductive analysis was conducted, where thematic categories were developed using Creswell et al. (2007) coding process.	The programme was perceived as effective in improving children’s PL. Specifically, the programme increased children’s involvement, engagement, confidence, sense of achievement, motor development, physical skills including coordination, social connectedness and cognitive skills. The factors associated with changes included the programme alignment to HPE curriculum, and programme elements closely aligned to PL domains.
Wright et al. (2020)	Explored participant experiences during a PL-focused JEPD programme, and perceived feasibility of the programme.	Elementary schools in British Columbia, Canada	*N* = 15 (87% female; 67% aged 25−44 years; 35% aged 45−64 years). Average years of experience was 14 years. 80% reported previous training on PA, 40% PL, 20% sedentary behaviours, 13% on other relevent topics. Most teachers received prep through a University course (87%), and taught children in a variety of grades between Kindergarten and Grade 5.	Pragmatic, feasibility trial with a quasi-experimental, mixed-methods design. Qualitative data was collected via post-programme, semi-structured interviews with participants. Transcripts were analysed using an inductive, constant comparison approach.	The programme impacted teacher’s practices including: implementation of more intentional activities of different variety and types, more student-focused discussions, enhanced transition activities, and adaptation of equipment. Factors associated with changes included the practicality and length of time of the programme, availability of resources and programme leader.

Note*.* PL = Physical Literacy; PA = Physical Activity; FMS = Fundamental Movement Skills; CT = Conventional Training; NT = No Training; ET = Exergame Training; SE-CPD = Sport-Education Professional Development; CP = Cerebral Palsy; PE = Physical Education; CG = Control Group; IG = Intervention Group; SES = Socio-Economic Status; PEPL = Physical Education Physical Literacy; MVPA = Moderate-to-Vigorous Physical Activity; SEWB = Social-emotional Well-Being; CS = Cognitive Skills; MLP = Munchkin Leagues Programme; NRL = National Rugby League; ECC = Early Childhood Centres; HPE = Health and Physical Education.

The included studies represented six countries: Australia (Clutterbuck et al., 2020; Telford et al., 2020a, 2020b; Usher, 2018), Canada (Bremer et al., 2020; Campelo & Katz, 2020; Wright et al., 2020), China (Choi et al., 2021), New Zealand (McLachlan et al., 2017), Portugal (Farias et al., 2020), and the United Kingdom (Edwards et al., 2018; Everley, 2021). Study designs varied, including interpretive descriptive, experimental mixed-methods, qualitative, and case study approaches, with qualitative methods such as semi-structured interviews (Choi et al., 2021; Edwards et al., 2018; McLachlan et al., 2017; Telford et al., 2020b; Usher, 2018; Wright et al., 2020), focus groups (Campelo & Katz, 2020; Everley, 2021; Farias et al., 2020; Telford et al., 2020a), and open-ended surveys (Clutterbuck et al., 2020; Farias et al., 2020; Wright et al., 2020). Two studies specifically reported qualitative results related to programme feasibility and acceptability (Bremer et al., 2020; Clutterbuck et al., 2020). Overall, the included studies focused on participant experiences in PL interventions, with some exploring specific topics such as older adults’ use of wearable technology in exergame programmes (Campelo & Katz, 2020). Interventions varied including sports/physical education programmes (Bremer et al., 2020; Choi et al., 2021; Clutterbuck et al., 2020; Everley, 2021; Farias et al., 2020; McLachlan et al., 2017; Telford et al., 2020a; Usher, 2018), and professional development for teachers (Edwards et al., 2018; McLachlan et al., 2017; Wright et al., 2020). Further details of the study characteristics are provided in Table II, and intervention-specific details can be found in the parent systematic review (Carl et al., 2022a).

### Synthesis findings

The level of credibility of each finding was *Unequivocal*, and is presented in a Summary of Findings (See Supplementary File). The meta-aggregation of participant experiences in PL interventions resulted in a total of three overarching findings: 1) Programme outcomes, 2) Factors influencing outcomes, and 3) Challenges with implementation, which are described in detail below.

#### Programme outcomes

**Figure 2.**
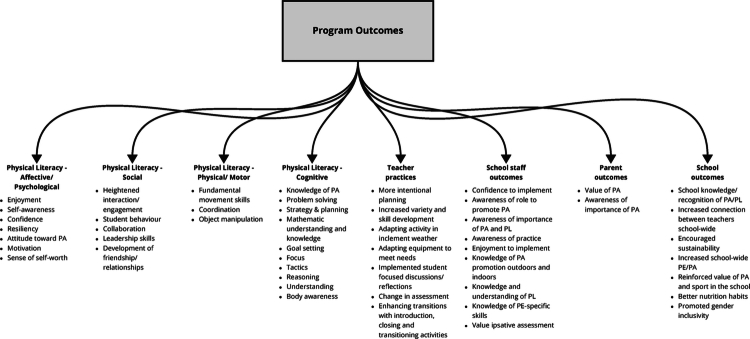
Visual hierarchy of Programme outcomes (grey-shaded box), their themes (bold) and sub-themes (bullet points) identified across PL interventions.

The analysis identified a total of eight themes within this overarching finding, where four related to the APLF domains of PL (i.e., affective/psychological capabilities, social capabilities, physical/motor capabilities, cognitive capabilities; ASC, 2025). The remaining related to educator practices, educator/principal characteristics, parent characteristics, and school outcomes. Figure 2 illustrates these themes including their corresponding sub-themes.

##### Physical literacy—affective/psychological capabilities.

PL interventions seemed to positively influence participants’ perceptions of their affective and psychological capabilities, including their enjoyment (Campelo & Katz, 2020; Clutterbuck et al., 2020; Telford et al., 2020a; Usher, 2018), self-awareness (Bremer et al., 2020; Farias et al., 2020; Everley, 2021; Clutterbuck et al., 2020; McLachlan et al., 2017; Telford et al., 2020a; Usher, 2018), confidence (Clutterbuck et al., 2020), and motivation (Everley, 2021), all in relation to PA. Additionally, the interventions seemed to foster resilience and self-worth, as highlighted by Farias et al. (2020), where one participant reflected, “In the sixth grade, I felt PE was a waste of time. Then in the seventh grade, I really started to like it,” (p. 271), further illustrating a shift in attitude towards PE.

##### Physical literacy—social capabilities.

The PL interventions also seemed to have a notable impact on participants’ social capabilities including their engagement (Bremer et al., 2020; Campelo & Katz, 2020; Choi et al., 2021; Clutterbuck et al., 2020; Farias et al 2020), collaboration (Bremer et al., 2020; Clutterbuck et al., 2020; Everley, 2021; Farias et al., 2020; Telford et al., 2020a; Usher 2018), behaviour (Clutterbuck et al., 2020; McLachlan et al., 2017; Telford et al 2020a), leadership (Bremer et al., 2020; Everley, 2021; Farias et al., 2020), and relationships (Everley, 2021; Farias et al., 2020). Heightened interaction was another key outcome, with one student reflecting, “Before we did this I never used to talk to people in my class. But now. I talk to other people. Before I used to be shy, but not anymore” (Telford et al., 2020a, *p*. 7). This increased interaction was accompanied by improvements in behaviour, as one parent from Clutterbuck et al. (2020) study noted, “[My son] learnt to be patient and understanding of others in the group. He became a more positive and happy soul, as part of a team” (p. 6).

##### Physical literacy—physical/motor capabilities.

Participants also reported experiencing improvements in their physical and motor capabilities, particularly their fundamental movement skills (Bremer et al., 2020; Clutterbuck et al., 2020; Edwards et al., 2018), coordination (Telford et al., 2020a; Usher, 2018), and object manipulation (Usher, 2018). Edwards et al. (2018), for example, found that “all teachers predominantly developed locomotor skills, [and] partly developed manipulative skills” (p. 130). Furthermore, one parent noted within Usher’s (2018) study that, “My daughter has improved her running and movement. She can pass and move around objects better and as a result her general sporting skill has improved” (p. 41).

##### Physical literacy—cognitive capabilities.

Intervention participants reported experiencing improvement in their cognitive capabilities such as increased awareness and knowledge of PA (Bremer et al., 2020; Campelo & Katz, 2020; Farias et al., 2020; Telford et al., 2020a), problem-solving (Everley, 2021; Telford et al., 2020a), and strategy and planning (Campelo & Katz, 2020; Clutterbuck et al., 2020; Everley, 2021; Farias et al., 2020; Telford et al., 2020a). To illustrate an improvement in knowledge of PA, one particpant from Farias et al. (2020) stated, “In the first units, I didn’t know the ‘shape of a ball’ (laughs). Then, I left the seventh-grade knowing how to support, pass, shoot” (p. 271).

Improvements were also made in mathematical understanding (Telford et al., 2020b; Usher, 2018), focus (Farias et al., 2020; Telford et al., 2020a), and tactical reasoning (Farias et al., 2020), with participants’ perceived demonstration better concentration, goal setting, and application of learned tactics in game situations (Farias et al., 2020; Telford et al., 2020a). Lastly, body awareness improved as well, with participants becoming more attuned to their physical capabilities (Campelo & Katz, 2020).

##### Teacher practices.

The PL interventions also seemed to influence teachers’ practices including how they plan, execute, and assess PE and PA (Edwards et al., 2018). Teachers from McLachlan et al.’s (2017) study reported being “more thoughtful and engaged” in planning activities (p. 233), while those in Telford et al.'s (2020b) study noted the “physical education and physical literacy **(**PEPL) approach’ allowed for better integration of sports into the classroom, linking it with other subjects. This shift was accompanied by increased variety in skill development, where teachers became more familiar with skill development and began incorporating game-oriented skills into their lessons (Wright et al. 2020), and lessons were enhanced with new structured transitions (e.g., warm-up; Wright et al., 2020). Additionally, teachers were able to show meaningful adaptations to barriers (e.g., inclement weather; McLachlan et al., 2017) and expand their use of equipment to meet diverse student needs, as one teacher noted, “[I’m] using more equipment that I had not considered before” (Wright et al., 2020, *p*. 12).

The interventions also revealed that student-focused discussions and reflections became a more prominent feature. Teachers in Wright et al.'s (2020) study for example, involved children in reflections using simple systems, such as the “silent thumbs up, thumbs down system” (p. 12), and encouraged reflective practices during games to provide better feedback to students.

##### School staff characteristics.

The PL interventions seemed to also improve school staff participants’ confidence (Edwards et al., 2018; McLachlan et al., 2017; Telford et al., 2020b), awareness of the importance of PA (Edwards et al., 2018), and enjoyment in promoting and implementing PA (Telford et al., 2020b). McLachlan et al. (2017) reported that most teachers felt more confident encouraging PA among young children, while teachers in Edwards et al. (2018) study reported they became more conscious of their role in fostering long-term health outcomes. One teacher noted, “We [teachers] instil enthusiasm in children. so when they become adults, there will be fewer problems with their health” (Edwards et al., 2018, *p*. 131). Teachers also enhanced their skills in using ipsative assessment to focus on individual progress, where one teacher stated, “It’s not about winning or who’s the best, it’s about improving on what they have done in the past” (Edwards et al., 2018, *p*. 132).

##### Parent outcomes.

Parents experienced benefits from the PL interventions as well, particularly in respect to their value for PA, and heightened awareness of its importance for their children. In Telford et al.'s (2020b) study, parents increasingly valued PA, as seen by changes such as bringing their children to school earlier to allow more time for PA; indicating a shift toward integrating PA into daily life. Furthermore, McLachlan et al. (2017) found that parents developed a deeper awareness of the importance of diet, PA, and motor skills for their child’s development.

##### School outcomes.

The PL interventions seemed to also lead to positive outcomes at the school level, including enhancing school knowledge and recognition of PA (Telford et al., 2020b), increased collaboration (Telford et al., 2020b), and sustained participation in and valuation of PA (Telford et al., 2020b). Schools also gained valuable resources and a deeper understanding of PA and PL (Telford et al., 2020b). Additionally, McLachlan et al. (2017) reported improved nutrition habits in early learning centres, while Bremer et al. (2020) highlighted the promotion of gender inclusivity, as one programme leader noted, “I think this was very helpful in increasing participant skills and increasing the idea of gender inclusivity [not all children fully understand this concept yet, but it is starting to set in]” (p. 13).

#### Factors influencing outcomes

**Figure 3.**
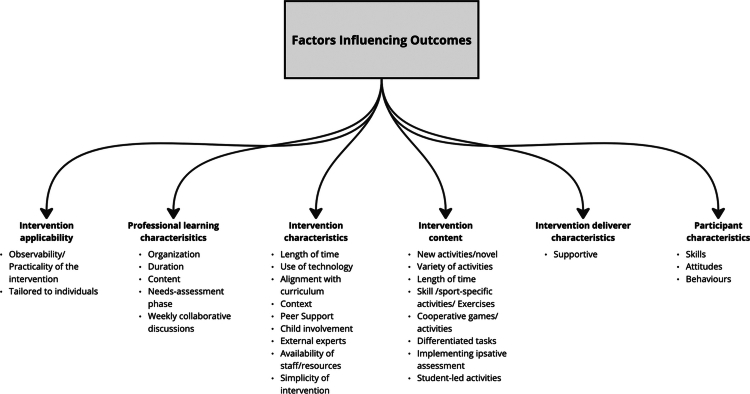
Visual hierarchy of Factors influencing changes (grey-shaded box), their themes (bold) and sub-themes (bullet points) identified across PL interventions.

Six key themes were identified as factors influencing the success of PL interventions: intervention applicability, intervention characteristics, professional learning characteristics, intervention content, intervention leader characteristics, and participant characteristics. Each theme is explored in more detail below and is illustrated in Figure 3.

##### Intervention applicability.

Teachers from Wright et al.'s (2020) study found that experiencing the interventions in action provided a clear understanding of how to implement the activities effectively. One participant specifically noted the advantage of observing the intervention in their own space, with their own students, which seemed to increase teachers’ confidence in implementation (Wright et al., 2020). The practical applicability extended to students as well, with one child participant in Telford et al. (2020a) noting, “I liked playing the different games because at home you can teach your family how to play it” (p. 7).

##### Professional learning characteristics.

Key aspects of professional learning, such as organisation (Wright et al., 2020), duration, and content (Choi et al., 2021; Edwards et al., 2018; McLachlan et al., 2017), significantly shaped the outcomes of PL interventions. Wright et al. (2020) emphasised the importance of well-structured sessions, with participants praising the clarity and thoughtful planning. Meanwhile, Choi et al. (2021) reported that coaching task cards were instrumental in shifting pedagogy toward a more student-centred approach. The needs-assessment phase in Edwards et al.'s (2018) study was also reported as influential in tailoring interventions to specific challenges, ensuring that learning environments catered to the variance in students’ abilities. Further, weekly collaborative discussions seemed to increase teachers’ PE-specific knowledge and awareness of their role in promoting health outcomes, leading to improvements in teaching practices (Edwards et al., 2018).

##### Intervention characteristics.

The characteristics of the PL interventions such as the use of technology (Campelo & Katz, 2020), alignment with the curriculum (Telford et al., 2020a; Wright et al., 2020), and context (e.g., outside clinical settings; Clutterbuck et al., 2020), were significant in delivering positive outcomes. Moreover, peer support (Bremer et al., 2020; Campelo & Katz, 2020; Choi et al., 2021; Clutterbuck et al., 2020; Everley, 2021; Farias et al., 2020; Telford et al., 2020a; Usher, 2018), child involvement, and external expertise were also pivotal in shaping outcomes (McLachlan et al., 2017). Farias et al. (2020) noted that longer intervention units contributed positively to their studies success, and availability of support or resources (e.g., contact with staff) during the interventions seemed to enhance participants’ outcomes (Bremer et al., 2020; Campelo & Katz, 2020; Wright et al., 2020).

##### Intervention content.

The content of PL interventions played an important role in shaping participants’ outcomes, with several key elements contributing to positive experiences such as novel activities (Bremer et al., 2020), cooperative games/activities (Campelo & Katz, 2020; Clutterbuck et al., 2020; Everley, 2021; Farias et al., 2020), and variety in activities was found critical in maintaining participant engagement (Bremer et al., 2020; Campelo & Katz, 2020).

Skill-specific exercises, such as volleyball drills, were instrumental in helping participants feel more capable over time (Farias et al., 2020), while cooperative games, like tee-ball, promoted team engagement (Clutterbuck et al., 2020). Similarly, differentiated tasks, ipsative assessment, and student-led activities further personalised the interventions (Edwards et al., 2018). For example, students in Edwards et al. (2018) study could choose their own level of ability, and student-led activities gave participants a sense of ownership. One student in Farias et al. (2020) study recalled, “In the seventh grade I really started to like it. that friendly competition, no teacher did that again in the eighth and ninth grades” (p. 271). These elements collectively ensured that the interventions were engaging, inclusive, and adapted to participants’ needs.

##### Intervention leader characteristics.

Characteristics of intervention leaders (i.e., facilitators) were highly influential in creating positive outcomes of the PL interventions, specifically being supportive (Campelo & Katz, 2020; Telford et al., 2020a, 2020b; McLachlan et al., 2017). This is made evident from participants in Campelo and Katz’s (2020) study who stated, “The staff members were just incredibly great to have in the room, because they were always supportive, and they were always there for us” (p. 6).

##### Participant characteristics.

Along with characteristics of leaders, the characteristics of participants themselves significantly influenced the outcomes of PL interventions. Having core skills already in place made it easier for subsequent lessons to build on that foundation (Telford et al., 2020a). Participants’ attitudes also played a crucial role, such as having a “just try” mindset helped them embrace new ideas and content (Telford et al., 2020a). Effective behaviour management further improved engagement, leading to more time on task and greater enjoyment for the participants (Telford et al., 2020a).

#### Challenges with implementation

**Figure 4.**
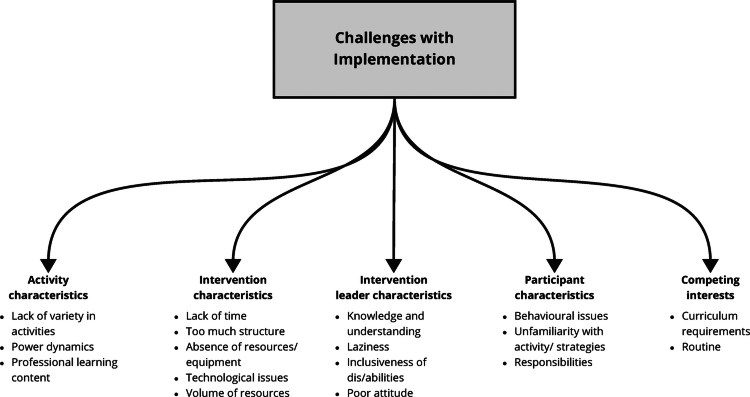
Visual hierarchy of challenges with implementation (grey-shaded box), their themes (bold) and sub-themes (bullet points) identified across PL interventions.

The analysis identified five main themes relating to perceived challenges in implementing the interventions: the characteristics of interventions and leaders, activity and participant characteristics, and competing interests. Each theme is comprised of several sub-themes, as shown in Figure 4 and described below.

##### Intervention characteristics.

One common issue was the lack of time to accommodate the interventions in participants’ schedules (Bremer et al., 2020). In some cases, participants found the structure of the programme too rigid, as described in Bremer et al. (2020), where one programme deliverer participant said, “I think structure is important though, and kids need structure, but it was too tight” (p. 13). The absence of resources also posed a problem. For instance, a participant in Campelo and Katz (2020) mentioned, “Half of the time I forgot the thing [Fitbit] was there… we didn’t have access to our accounts, and we had to come only once a week” (p. 6). Technological issues were additional barriers to effective implementation, and the volume of new content and activities to integrate were found to be overwhelming (Wright et al., 2020).

##### Intervention leader characteristics.

Participants faced challenges related to the characteristics of the intervention leaders during the PL programmes. A lack of enthusiasm or perceived laziness from leaders also negatively impacted the experience, as noted in Bremer et al. (2020), where a participant stated that their least favourite part was “keeping children engaged in repeat games/activities and having to keep up engagement” (p. 13). This sentiment was echoed in Farias et al.'s (2020) study, where one participant remarked, “I guess sometimes I didn’t put my best effort in it. The teacher didn’t care, why should I?” (p. 273). Such poor attitudes from leaders led to a sense of disengagement, undermining the effectiveness of the intervention. Further, leaders’ knowledge and understanding of how to accommodate diverse needs and age groups were identified as areas needing improvement (Wright et al., 2020).

##### Activity characteristics.

Intervention participants (e.g., programme deliverers) also encountered challenges related to the nature of intervention activities, which affected both engagement and overall programme success, with the lack of variety in activities marked as a common concern. Bremer et al. (2020) reported that participants suggested, “An increase in the variety of activities would be helpful in keeping children engaged” (p. 13), indicating that repetitive activities decreased their interest. Power dynamics within activities also affected participants’ experiences (Choi et al., 2021; Everley, 2021; Farias et al., 2020). In Farias et al.'s (2020) study, one child mentioned, “At first, the leaders didn’t let us choose the games we wanted to play” (p. 272), which limited their autonomy and affected their motivation. Lastly, some participants struggled with the professional learning content, as noted by Choi et al. (2021), where lecturers felt that the two-dimensional representation of coaching task cards could lead to interpretational issues, complicating the understanding and implementation of activities.

##### Participant characteristics.

A range of participant characteristics posed as challenges during the implementation of PL interventions. Behavioural issues seemed to complicate implementation, with Bremer et al. (2020) observing that some children “wanted to do their own thing” (p. 13), making group cohesion difficult to achieve.

Unfamiliarity with the assigned activities or roles also disrupted participation. Choi et al. (2021) highlighted how a lack of understanding often led to uneven workloads within teams, causing frustration and disengagement among participants. Additionally, a sense of being overwhelmed by responsibilities detracted from the overall experience (Choi et al., 2021; Farias et al., 2020). One participant in Farias et al. (2020) shared, “sometimes, having the feeling of being a bit “fenced”. Always responsible for something” (p. 271) made the activities feel less enjoyable and more burdensome. Another participant from Choi et al.'s (2021) study echoed this, explaining how the dual role of student-coaches made them miss warm-ups, thus limiting their participation in key aspects of the activities.

#### Competing interests

A key challenge participants encountered during the PL interventions was balancing competing interests, particularly curriculum requirements (Bremer et al., 2020; Choi et al., 2021; Edwards et al., 2018; McLachlan et al., 2017; Wright et al., 2020) and established routines (Bremer et al., 2020; McLachlan et al., 2017). McLachlan et al. (2017) highlighted the need for a stronger connection between the interventions and the curriculum, with participants suggesting that future professional learning should focus more on the links to New Zealand’s early years curriculum, Te Whāriki. As well, some participants from McLachlan et al.'s (2017) study felt that having “a concrete learning outcome for physical education” (p. 223) would help teachers align their efforts and focus on the same developmental goals.

Routine disruptions also added to the difficulty of implementing the interventions. As noted in Wright et al. (2020), participants found mid-year changes challenging, with one stating that adjustments made “mid-year rather than at the beginning in September” (p. 13) were harder to incorporate into planned schedules. Furthermore, Bremer et al. (2020) observed that highly structured programmes could be integrated more easily into routines, while less structured programmes were more difficult to manage.

## Discussion

This synthesis provided new insights into the perceived outcomes as a result of PL interventions, participants’ experiences during PL interventions, and the factors which affected the outcomes reported. These overarching findings emphasised the multi-dimensional impact of PL interventions, not only on participants’ broader well-being and sense of self, but at levels in the environment (e.g., school climate; Telford et al., 2020a, 2020b). By synthesising qualitative data from diverse settings and demographic groups, this study reinforced the necessity of considering PL as a holistic construct and contributes to advancing intervention strategies that can foster lifelong PA participation.

The meta-aggregation revealed significant positive outcomes across the physical, psychological, social, and cognitive domains of PL, aligning with ASC (2025) APLF framework. Participants across studies reported enhanced confidence, engagement, and resilience, suggesting that interventions effectively addressed psychological barriers to PA. These outcomes were complemented by improved motor, cognitive, and social capabilities, which collectively promoted meaningful engagement in PA. Importantly, these findings extended a previous literature review (Carl et al., 2022b) by emphasising the nuanced ways in which PL programmes influence individual experiences and development, providing a deeper understanding of the lived realities of participants.

Similar patterns, also gathered through qualitative syntheses, are reported in other fields. For example, Daly-Smith et al., (2021) found that physically active learning is more likely to be adopted and maintained when programmes promote teachers’ confidence, supply practical resources, and are supported by the whole school. Further, Costes-Onishi et al. (2020) showed that inquiry-based lessons in the arts and humanities produce the strongest academic and social gains when activities are hands-on and encourage peer collaboration. These cross-field insights, similar to our findings, highlight the need to attend to both psychological factors and social context when designing movement-centred interventions.

Several factors were identified as pivotal in shaping the success of PL interventions, including the adaptability of programme content and characteristics of intervention leaders and programme facilitators. For instance, Wright et al. (2020) conducted a needs-assessment phase with educators and found that schools that co-designed activities during this phase reported higher teacher uptake and clearer curriculum alignment than those that adopted the generic lesson plans.

The data highlighted the importance of professional development for educators at all levels of education (Choi et al., 2021; McLachlan et al., 2017; Telford et al., 2020a, 2020b; Wright et al., 2020), as well as tailored interventions that align with specific community needs (Campelo & Katz, 2020). These findings supported prior calls for context-sensitive approaches to PL (Caldwell et al., 2020; Mohammadi et al., 2023), as PL interventions that are rigid or inadequately supported by facilitators risk alienating participants and reducing programme efficacy.

Despite these positive outcomes, challenges such as limited resources, time constraints, and competing priorities were recurrent themes across the included studies (e.g., McLachlan et al., 2017; Wright et al., 2020). These insights suggest opportunities for future interventions to integrate innovative and adaptable strategies, such as leveraging technology or incorporating community-based participatory approaches.

### Practical considerations

The evidence shows that physical literacy programmes have the strongest impact when they nurture physical, cognitive, social, and affective growth together (Cairney et al., 2019). Practitioners can apply this insight in four concrete ways. First, curricula should integrate activities that touch every domain. An example of this is School of Sport Education Northern Territory’s (2021) *Body–Brain–Being: Active Learning Plan—Transition–Year 2*. This resource illustrates this approach by embedding movement into literacy, numeracy, and enquiry lessons while pairing each task with confidence-building and cooperation goals. Second, professional learning must be ongoing and classroom-based. From our findings, teachers who received in-situ mentoring and peer support adopted PL practices more consistently and reported better child outcomes than those who attended one-off workshops (Edwards et al., 2018; Wright et al., 2020). Third, programmes should be co-designed with children, families, and educators. Our results demonstrated interventions tailored to local interests and schedules achieved higher engagement and more durable behaviour change than standardised programmes (Edwards et al., 2018; Telford et al., 2020a). Finally, progress monitoring should rely on formative, ipsative tools that highlight individual improvement rather than only age norms (Carl et al., 2024; Pushkarenko et al., 2023). Aligning policy, training, and daily practice with these principles can help PL interventions foster lifelong participation in PA.

### Strengths and limitations

One key strength of our study lies in the use of a qualitative meta-synthesis approach, which is relatively uncommon in PL intervention research (Carl et al., 2022a). Most studies in the field focus primarily on measuring quantitative outcomes, such as improvements in physical competence, motivation, or activity levels (Edwards et al., 2018). While quantitative data provides important evidence of intervention effectiveness, the qualitative insights offered in this study reveal deeper understanding about the lived experiences of participants, the challenges they face, and the contextual factors that influence the success of PL interventions (Leavy, 2017). As previously noted, qualitative research is essential for uncovering the nuanced experiences of individuals engaged in PL interventions, which may be overlooked in purely quantitative studies (Edwards et al., 2019). By capturing these nuanced experiences, our study offers rich insights into the psychological and social dimensions of PL that are often neglected in more quantitative-focused evaluations (Edwards et al., 2018).

One key limitation of our qualitative synthesis is that it is bounded by the scope of a single, previously published systematic review of PL interventions (Carl et al., 2022a). As such, only studies included in that review and published between 2017 and 2022 were eligible for inclusion. This reliance means that relevant qualitative or mixed-methods intervention studies falling outside its search strategy, time frame, or inclusion criteria may not be represented in our synthesis, which subsequently impacts the breadth of our results.

The included studies represented six predominantly high-income countries: Australia (Clutterbuck et al., 2020; Telford et al., 2020a, 2020b; Usher, 2018), Canada (Bremer et al., 2020; Campelo & Katz, 2020; Wright et al., 2020), China (Choi et al., 2021), New Zealand (McLachlan et al., 2017), Portugal (Farias et al., 2020), and the United Kingdom (Edwards et al., 2018; Everley, 2021). This geographical concentration suggests that qualitative evaluations of PL interventions are currently more established in these contexts, whereas other countries may have prioritised quantitative outcome measurement. As a result, the transferability of our findings to low- and middle-income countries or non-Anglophone contexts should be interpreted with caution.

We also acknowledge that PL intervention research continues to evolve beyond the 2017–2022 period captured in the parent systematic review. Emerging studies published after 2022, including additional mixed-methods and qualitative evaluations of PL programmes, may offer further insight into participant experiences, implementation processes, and context-specific outcomes. While some of these newer studies did not meet our inclusion criteria (e.g., absence of extractable qualitative data focused on PL outcomes), a future review that updates and extends the search beyond the 2022 cut-off would be valuable to determine whether our themes remain stable or are augmented by more recent work.

The limitations of the meta-aggregation approach should also be acknowledged. While meta-aggregation is a powerful method for synthesising qualitative findings, it can sometimes reduce the complexity and richness of individual studies (Bergdahl, 2019). However, as Lockwood et al. (2015) note, meta-aggregation provides a reliable and consistent method for reviewing large bodies of qualitative data, and it ensures that key themes and findings are synthesised in a way that supports practical recommendations. In the context of PL interventions, this approach allowed us to consolidate diverse qualitative findings into coherent insights that can guide future intervention development.

## Conclusions

In conclusion, this study highlights holistic and context-sensitive PL interventions that address the multifaceted nature of PL. By integrating physical, cognitive, social, and psychological domains, these interventions can support individuals across the lifespan in developing the capabilities needed to engage in lifelong PA. The qualitative nature of our research has added significant depth to this understanding, revealing how participants experience PL interventions and providing insights into the factors that influence their success. These participant-centred practices also have measurement implications; assessment tools should reflect what learners value and avoid reducing PL to easily observed behaviours (Durden-Myers et al., 2022; Pot et al., 2018). Barnett et al.'s (2019) nine-step guide offers a pragmatic way to combine objective skill cheques with participant-led reflections, ensuring evaluation remains aligned with programme goals and context.

Future research should continue to refine PL interventions, ensuring that they are theory-based (Carl et al., 2022a), well-supported by cross-sectoral collaborations (Pratt et al., 2020), and tailored to the diverse needs of populations, particularly in low- and middle-income countries (Pratt et al., 2023). As PL interventions evolve, it is essential to adopt standardised reporting frameworks like the Physical Literacy Intervention Reporting Template (PLIRT; Carl, Bryant, et al., 2023) to improve transparency, scalability, and replication across studies. Ultimately, these recommendations can help bridge the gap between theory and practice to promote lifelong PA, an enormously important public health priority (Pratt et al., 2020; Pratt et al., 2023), and enhance the effectiveness of PL interventions globally.

## Supplementary Material

Suppl File.xlsx Suppl File.xlsx

PRISMA_2020_checklist.pdf PRISMA_2020_checklist.pdf

## Data Availability

The authors confirm that the data supporting the findings of this study are available within the article and its supplementary materials.
